# CtaM Is Required for Menaquinol Oxidase *aa*_3_ Function in *Staphylococcus aureus*

**DOI:** 10.1128/mBio.00823-16

**Published:** 2016-07-12

**Authors:** Neal D. Hammer, Lici A. Schurig-Briccio, Svetlana Y. Gerdes, Robert B. Gennis, Eric P. Skaar

**Affiliations:** a Department of Microbiology and Molecular Genetics, Michigan State University, East Lansing, Michigan, USA; b Department of Biochemistry, University of Illinois, Urbana, Illinois, USA; c Fellowship for Interpretation of Genomes, Burr Ridge, Illinois, USA; d Department of Pathology, Microbiology, and Immunology, Vanderbilt University Medical Center, Nashville, Tennessee, USA

## Abstract

*Staphylococcus aureus* is the leading cause of skin and soft tissue infections, bacteremia, osteomyelitis, and endocarditis in the developed world. The ability of *S. aureus* to cause substantial disease in distinct host environments is supported by a flexible metabolism that allows this pathogen to overcome challenges unique to each host organ. One feature of staphylococcal metabolic flexibility is a branched aerobic respiratory chain composed of multiple terminal oxidases. Whereas previous biochemical and spectroscopic studies reported the presence of three different respiratory oxygen reductases (*o* type, *bd* type, and *aa*_3_ type), the genome contains genes encoding only two respiratory oxygen reductases, *cydAB* and *qoxABCD*. Previous investigation showed that *cydAB* and *qoxABCD* are required to colonize specific host organs, the murine heart and liver, respectively. This work seeks to clarify the relationship between the genetic studies showing the unique roles of the *cydAB* and *qoxABCD* in virulence and the respiratory reductases reported in the literature. We establish that QoxABCD is an *aa*_3_-type menaquinol oxidase but that this enzyme is promiscuous in that it can assemble as a *bo*_3_-type menaquinol oxidase. However, the *bo*_3_ form of QoxABCD restricts the carbon sources that can support the growth of *S. aureus*. In addition, QoxABCD function is supported by a previously uncharacterized protein, which we have named CtaM, that is conserved in aerobically respiring *Firmicutes*. In total, these studies establish the heme A biosynthesis pathway in *S. aureus*, determine that QoxABCD is a type *aa*_3_ menaquinol oxidase, and reveal CtaM as a new protein required for type *aa*_3_ menaquinol oxidase function in multiple bacterial genera.

## IMPORTANCE

*Staphylococcus aureus* relies upon the function of two terminal oxidases, CydAB and QoxABCD, to aerobically respire and colonize distinct host tissues. Previous biochemical studies support the conclusion that a third terminal oxidase is also present. We establish the components of the *S. aureus* electron transport chain by determining the heme cofactors that interact with QoxABCD. This insight explains previous observations by revealing that QoxABCD can utilize different heme cofactors and confirms that the electron transport chain of *S. aureus* is comprised of two terminal menaquinol oxidases. In addition, a newly identified protein, CtaM, is found to be required for the function of QoxABCD. These results provide a more complete assessment of the molecular mechanisms that support staphylococcal respiration.

## INTRODUCTION

*Staphylococcus aureus* is a significant cause of morbidity and mortality worldwide (1). In the United States, *S. aureus* is the leading cause of skin and soft tissue infections, endocarditis, septicemia, and osteomyelitis (1, 2). In order to colonize these diverse niches, *S. aureus* relies upon a dynamic metabolism that supports proliferation by overcoming the unique challenges presented by the host innate immune response (3–5). *S. aureus* utilizes three energy-generating pathways to support proliferation: aerobic respiration, fermentation, and anaerobic respiration (6–8). Previous reports demonstrate that aerobic respiration is required for colonization of the heart and liver (3, 4). The enzymes that catalyze the final reaction of aerobic respiration, the reduction of oxygen to water, are called terminal oxidases (9, 10). Most bacteria encode more than one terminal oxidase in their genome, with expression dependent on the growth conditions. This allows an organism to adapt its respiratory chain to changes in its environment, such as the oxygen concentration. The *S. aureus* genome encodes two terminal oxidases, *cydAB* and *qoxABCD* (3). This branched respiratory chain supports organ-specific colonization by *S. aureus*, as mutants with mutations that inactivate *cydAB* are unable to colonize the murine heart and impairment of *qoxABCD* dramatically decreases the capacity to infect the liver (3, 4).

In *S. aureus*, inactivation of *cydAB* together with *qoxABCD* arrests respiration and induces the small colony variant (SCV) phenotype (3, 11). This finding supports the conclusion that CydAB and QoxABCD are the exclusive terminal oxidases that power aerobic respiration in this pathogen. CydAB and QoxABCD are representatives of the two major superfamilies of respiratory oxygen reductases. *cydAB* encodes a *bd*-type menaquinol oxidase (9, 12), and *qoxABCD* encodes a menaquinol oxidase that is a member of the heme-copper oxygen reductase superfamily (13). Prior to the availability of the genome sequence of *S. aureus*, biochemical studies indicated that there were *o*-type and/or *aa*_3_-type terminal oxidases in the organism, but there is no mention of cytochrome *bd* (14–18). Typically, cytochrome *bd* can be identified spectroscopically due to the presence of heme D and by the resistance of the enzyme to cyanide (9). In fact, cytochrome *bd* cyanide resistance is a feature that distinguishes *S. aureus* from nonpathogenic staphylococci; *S. aureus* cytochrome *bd* is more sensitive to cyanide than the cytochrome *bd* expressed by the avirulent *Staphylococcus carnosus* (19). It was not until 2014 that the spectroscopic signature of *S. aureus* cytochrome *bd* was reported (20). Taken together, there is biochemical evidence for three types of respiratory oxygen reductases in *S. aureus*, a *bd* type, an *o* type, and/or an *aa*_3_ type, despite genomic evidence for only *cydAB* and *qoxABCD*. Here, we address the origin of the three putative terminal oxidases and delineate the cyanide sensitivity of the *S. aureus* cytochrome *bd*. It is shown that the *S. aureus* cytochrome *bd* is resistant to cyanide below 1 mM and that the QoxABCD enzyme exists in two forms. One form contains two equivalents of heme A (*aa*_3_ type), and the other incorporates hemes B and O (*bo*_3_ type). Both forms are functional and sensitive to cyanide. We also identify a new gene in the *S. aureus* genome that is conserved in many *Firmicutes* and is required for the assembly of functional QoxABCD in any form. This gene (locus NWMN_0982), which we name *ctaM*, is adjacent to the gene encoding the heme O synthase, *ctaB*. These results define the molecular requirements for terminal oxidase function in *S. aureus*, provide an explanation for the previous observations describing the presence of an *o*-type reductase in the staphylococcal respiratory chain, and establish a conserved protein involved in terminal oxidase assembly.

## RESULTS

### QoxABCD is an *aa*_3_-type terminal oxidase and CydAB is a canonical cyanide-resistant cytochrome *bd*.

**FIG 1:**
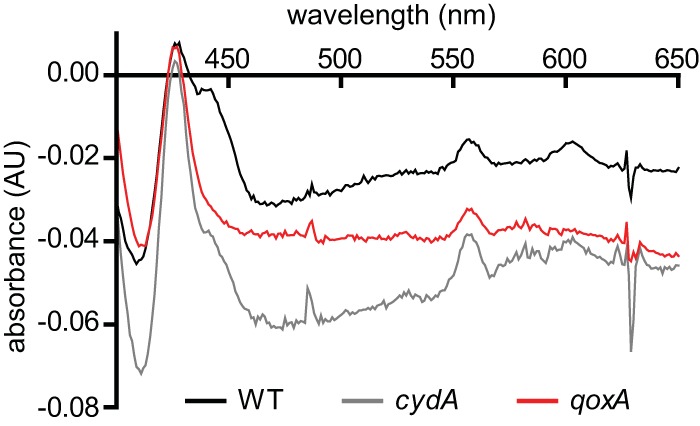
The cytochrome profiles of terminal oxidase mutants reveal the preferred heme cofactors utilized by each enzyme. The reduced-minus-oxidized spectra of *Staphylococcus aureus* and isogenic terminal oxidase mutants (Δ*cydA* and Δ*qoxA*) are shown. Heme A peaks are located at 430 nm and 609 nm. AU, arbitrary units.

The composition of the aerobic respiratory chain depends upon both the growth medium and the phase of growth at which the cells are harvested. Table 1 shows the comparison of the properties of membranes isolated from the wild type (WT) and different mutants of *S. aureus*, each grown in LB with 0.5% glucose and harvested after 8 h. The rate of O_2_ consumption (per mg of membrane protein) in the presence of NADH was measured in the presence and absence of 100 µM potassium cyanide (KCN). About 40% of the O_2_ consumption is inhibited by 100 µM KCN in the WT, whereas in the Δ*cydA* mutant, 100% inhibition is observed. In contrast, in the Δ*qoxA* mutant, 100% of the activity remains in the presence of 100 µM KCN. Hence, the oxygen reductase encoded by *cydAB* is cyanide resistant and the oxygen reductase encoded by *qoxABCD* is cyanide sensitive. The reduced-minus-oxidized difference spectra of the membranes from both the WT and Δ*cydA* strains (Fig. 1) clearly show the spectroscopic features of an *aa*_3_-type oxygen reductase (peaks at about 443 nm and 606 nm), which are absent from the spectrum of the Δ*qoxABCD* strain. The spectroscopic signature typical of heme D, a peak in the reduced-minus-oxidized spectrum near 630 nm, is not evident in any of the spectra in Fig. 1. This feature was previously observed with membranes from the WT strain, as shown in Fig. S4 in reference 20, so its absence is likely due to its relatively small amount and the noise in the spectra shown in Fig. 1. These results establish that *qoxABCD* encodes a cyanide-sensitive, *a*-type reductase.

**TABLE 1:** Respiratory activities of *S. aureus* terminal oxidase assembly mutants

Strain	Terminal oxidase(s) present	Membrane respiratory activity	
		After addition of 100 µM KCN (%)	Relative to that of WT
WT	*aa*_3_ and *bd*	40	1.0
Δ*cydA* mutant	*aa*_3_	0	0.8
Δ*qoxA* mutant	*bd*	100	1.1
Δ*ctaA* mutant	*bo*_3_ and *bd*	32	1.0
Δ*ctaB* mutant	*bd*	95	1.0
Δ*ctaM* mutant	*bd*	98	0.9
Δ*cydAB* Δ*ctaA* mutant	*bo*_3_	7	0.5
Δ*qoxB* Δ*ctaA* mutant	*bd*	96	1.3

### QoxABCD assembles as a functional oxidase in the absence of heme A.

**FIG 2:**
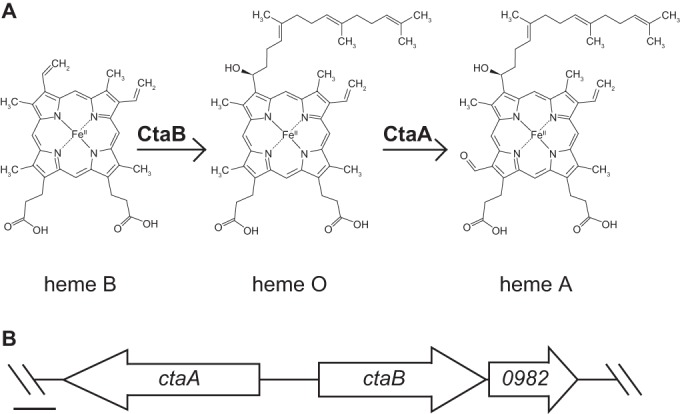
CtaB is predicted to be the heme O synthase in *S. aureus*. (A) Putative heme cofactor biosynthesis pathway in *S. aureus*. CtaA is the heme A synthase in *S. aureus* (21). (B) Genomic locus containing the heme cofactor biosynthesis genes in *S. aureus* strain Newman. 0982 denotes the locus tag NWMN_0982 (referred to herein as *ctaM*). Scale bar = 250 bp.

It is expected that the *aa*_3_-type menaquinol oxidase encoded by *qoxABCD* incorporates two equivalents of heme A. The conversion of heme B to heme A is catalyzed by CtaB and CtaA, which have been characterized from other bacteria, including *Bacillus subtilis* (21, 22). The reaction sequence is shown in Fig. 2A, showing heme B converted to heme O by CtaB and then to heme A via CtaA. Both the *ctaB* and *ctaA* genes are present in *S. aureus* and, in addition, there is a third gene adjacent to *ctaB* which we have named *ctaM* (locus NWMN_0982), whose function is not known (Fig. 2B).

**FIG 3:**
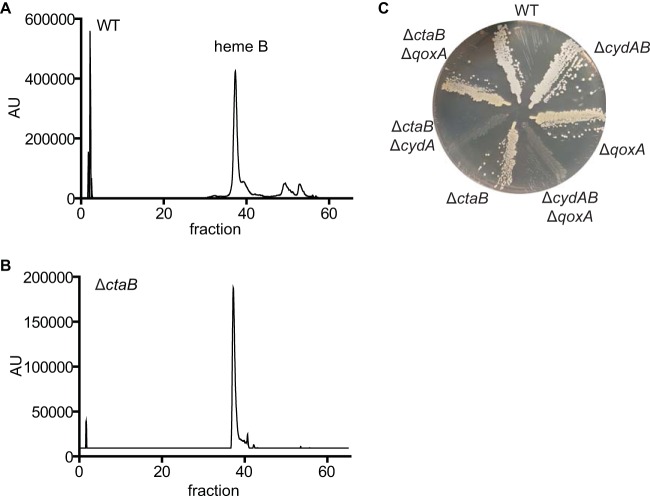
Heme O is synthesized by CtaB and is required for QoxABCD function. (A) Liquid chromatography (LC) analysis of membranes isolated from wild-type (WT) staphylococci. Tandem mass spectroscopy was used to identify heme B in fraction 37. (B) LC analysis of membranes isolated from an isogenic Δ*ctaB* deletion mutant. (C) Overnight cultures of WT *S. aureus* and isogenic mutants were grown at 37°C in tryptic soy broth (TSB) and streaked for isolated colonies on tryptic soy agar (TSA). The Δ*cydAB* Δ*qoxA* and Δ*ctaB* Δ*cydA* mutants are respiration-arrested small colony variants.

A mutant with a deletion of *ctaB* was constructed, and the Δ*ctaB* strain behaves like the Δ*qoxA* strain insofar as the remaining respiratory activity is entirely resistant to the presence of 100 µM KCN (Table 1). Figure 3A shows a liquid chromatography-mass spectrometry (LC-MS) analysis of the hemes extracted from the membranes of the WT strain, excluding heme D, which is not observed under these conditions. The major peak is due to heme B, expected to be a component of cytochrome *bd*, as well as succinate dehydrogenase. Two smaller peaks elute at positions indicating that they represent heme O and heme A. This is consistent with the analysis of the hemes present in the membranes of the Δ*ctaB* strain, in which these two small peaks are absent (Fig. 3B). In addition, the double Δ*ctaB* Δ*cydA* mutant exhibits the SCV phenotype (Fig. 3C), indicating that this strain is respiration deficient. These results demonstrate that the synthesis of heme O is essential for the assembly of functional QoxABCD.

**FIG 4:**
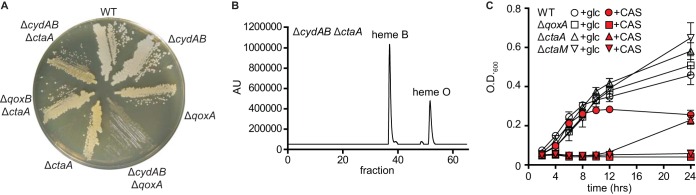
In the absence of heme A synthesis, QoxABCD utilizes heme O. (A) Overnight cultures of WT *S. aureus* and isogenic mutants were grown at 37°C in TSB and streaked for isolated colonies on TSA. The Δ*cydAB* Δ*qoxA* mutant is a respiration-arrested small colony variant. (B) LC analysis of membranes isolated from a Δ*cydAB* Δ*ctaA* mutant. Tandem mass spectroscopy was used to identify heme B in fraction 37 and heme O in fraction 52. (C) The growth of the WT and isogenic mutants of *S. aureus* was monitored over time by optical density at 600 nm. The final concentrations of glucose (glc) and Casamino Acids (CAS) added to the growth medium were 25 mM (open symbols) and 0.5% (red symbols), respectively. The average results from three independent experiments are shown. Error bars represent one standard deviation from the mean.

Unexpectedly, the Δ*ctaA* strain, which also cannot synthesize heme A (Fig. 2) (21), has 32% of its respiratory activity inhibited by 100 µM KCN (Table 1), indicating that a cyanide-sensitive terminal oxidase is present, similar to what is observed in the WT strain. The Δ*ctaA* Δ*cydAB* double mutant does not exhibit the SCV phenotype (Fig. 4A) and is not respiration deficient. Furthermore, the respiratory activity of the Δ*ctaA* Δ*cydAB* double mutant is virtually entirely inhibited by 100 µM KCN (Table 1). Therefore, blocking the conversion of heme O to heme A (Fig. 2) does not prevent QoxABCD from assembling to an active, cyanide-sensitive oxygen reductase. This result implies that QoxABCD can assemble in the absence of heme A by incorporating heme B and heme O. LC-MS analysis of the hemes extracted from membranes from the Δ*ctaA* strain shows a large peak identified as heme O, supporting this supposition (Fig. 4B). Previous studies on *Bacillus cereus* (23, 24) showed that preventing the synthesis of heme A results in the assembly of the *aa*_3_-type menaquinol oxidase into a *bo*_3_-type menaquinol oxidase. Our observations indicate that *S. aureus* responds in a similar manner.

### QoxABCD utilizes heme O at the expense of carbon restriction.

The ability of *B. subtilis* to grow in different media is impacted by mutations that eliminate one or more of the respiratory oxygen reductases (25). To determine whether the inability to synthesize heme A leads to any growth phenotype in *S. aureus*, WT, Δ*qoxA*, and Δ*ctaA* strains were grown in a minimal medium supplemented with glucose or Casamino Acids (CAS) as the carbon source (Fig. 4C). When glucose is supplied as a carbon source, the WT, Δ*ctaA*, and Δ*qoxA* strains grow equally well (Fig. 4C). However, when the cells are cultured in a minimal medium supplemented with CAS, no growth of the Δ*qoxA* strain is observed after 24 h and the growth of the Δ*ctaA* strain exhibits a lag of at least 12 h before growth is observed (Fig. 4C).

### CtaM is required for QoxABCD function.

**FIG 5:**
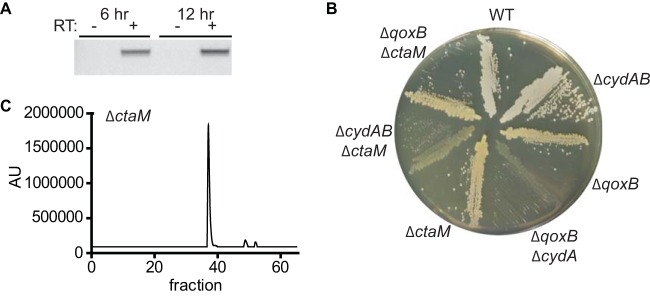
QoxABCD function is supported by CtaM. (A) PCR was performed on cDNA enriched from staphylococci grown at 37°C to early stationary (6 h) or late stationary (12 h) phase. RT, reverse transcriptase. (B) Overnight cultures of WT *S. aureus* and isogenic mutants were grown at 37°C in TSB and streaked for isolated colonies on TSA. The Δ*qoxB* Δ*cydA* and Δ*cydAB* Δ*ctaM* double mutants are respiration-arrested small colony variants. (C) LC analysis of membranes isolated from the Δ*ctaM* strain.

The gene adjacent to *ctaB*, NWMN_0982 (locus tag in *S. aureus* strain Newman), encodes a putative membrane protein of unknown function (Fig. 2B). The location adjacent to *ctaB* suggests that NWMN_0982 also facilitates QoxABCD function and that these genes are likely cotranscribed. To verify this, primers were designed to amplify between the coding regions of *ctaB* and NWMN_0982. Amplification between *ctaB* and NWMN_0982 was observed when cDNA was prepared from *S. aureus* grown to stationary phase and was dependent on reverse transcriptase (Fig. 5A). The finding that *ctaB* and NWMN_0982 are cotranscribed demonstrates that these genes reside in an operon and supports the hypothesis that these genes encode proteins that function in the same pathway. Consistent with this, we refer to NWMN_0982 as *ctaM* (cytochrome assembly). To test the hypothesis that *ctaM* supports QoxABCD function, *ctaM* was inactivated in the Δ*cydAB* strain. The Δ*cydAB* Δ*ctaM* mutant displays the SCV phenotype (Fig. 5B), and a plasmid-encoded copy of *ctaM* complements the respiration-arrested growth of the Δ*cydAB* Δ*ctaM* strain (see Fig. S2B in the supplemental material).

These results demonstrate that CtaM is required for the assembly of functional QoxABCD. Accordingly, the Δ*qoxB* Δ*ctaM* double mutant does not exhibit the SCV phenotype, indicating that CtaM is not required for CydAB activity (Fig. 5B). Consistent with this, the respiratory activity of the Δ*ctaM* strain is resistant to KCN, revealing that CydAB is the only terminal oxidase active in this mutant (Table 1). Furthermore, the Δ*ctaM* strain behaves like the Δ*qoxA* strain in that neither strain exhibits growth over a 24-h period in liquid broth consisting of a minimal medium supplemented with CAS (Fig. 4C). The hemes extracted from membranes prepared from the Δ*ctaM* strain are similar to the hemes isolated from the WT, showing a predominant LC peak due to heme B and small peaks that appear to correspond to heme O and heme A (Fig. 5C). These results suggest that CtaM is not required for the function of CtaA or CtaB but, rather, is needed either for the insertion of the hemes into the enzyme or for the activity of the final, assembled enzyme.

### *ctaM* is conserved in respiring *Firmicutes* that synthesize heme O and heme A.

**FIG 6:**
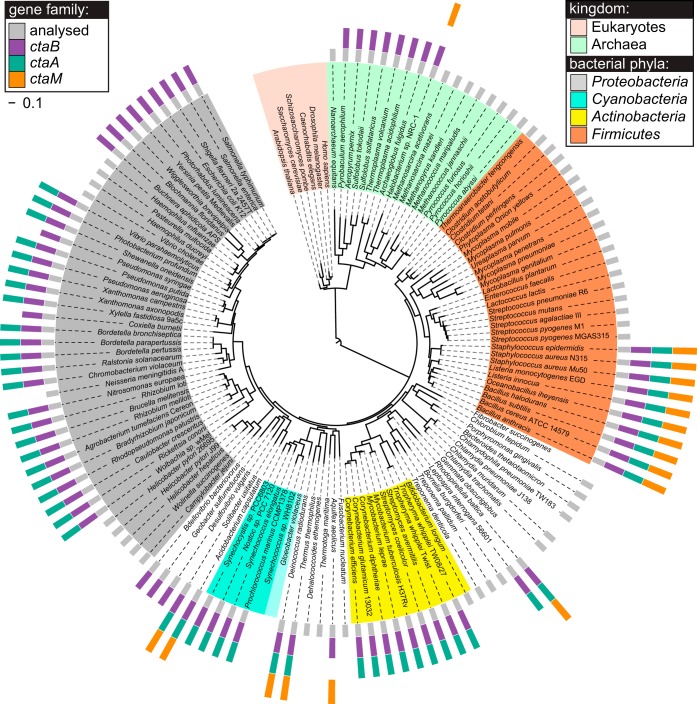
*ctaB*, *ctaA*, and *ctaM* homologues are conserved among respiring *Firmicutes* and show a strong tendency to co-occur in the same genome. The phylogenic distribution of *ctaM* (orange, outer ring) was mapped onto the Tree of Life (29, 45). The distributions of *ctaB*, the gene that encodes the heme O synthase (purple, outer ring), and of *ctaA*, the gene that encodes the heme A synthase (teal, outer ring), are also presented.

These results establish that *ctaM* is required for QoxABCD terminal oxidase function and imply that *ctaM* is conserved in other species of bacteria that utilize QoxABCD to respire. Phylogenic investigation in the SEED database (26, 27) determined that *ctaM* is conserved in many diverse species of bacteria, as the analysis of a set of 978 nonredundant representative prokaryotic genomes reveals a *ctaM* homologue in 120 organisms (12% of the genomes analyzed). The results of this analysis are illustrated in Fig. 6 and are available in full online in the SEED Subsystem “Heme O and Heme A biosynthesis (with selected terminal oxidases)” (28).

The presence of *ctaM* in the *Archaea*, *Firmicutes*, *Aquificae*, and *Planctomycetes*, some of the oldest evolutionary taxa, supports the conclusion that CtaM is an ancient protein. Consistent with this, *ctaM* is not present in *Beta*- or *Gammaproteobacteria* (the youngest taxa) but does occur in a few representatives of the *Alpha*- and *Delta*-/*Epsilonproteobacteria* (the older subgroups of *Proteobacteria*) (29). Notably, within each taxon, CtaM proteins are found only in genomes encoding *aa*_3_-type terminal oxidases and never in nonrespiring organisms or organisms utilizing other types of terminal oxidases (28). In particular, within the *Firmicutes*, *ctaM* is conserved among those that respire but not in obligate anaerobes like the *Clostridiales* or aerotolerant nonrespiring bacteria like the lactic acid bacteria (LAB). In some cases, when grown in medium supplemented with the proper cofactors, LAB respire by utilizing *bd*-type oxidase (CydAB) (30). This finding supports the idea that CtaM specifically interacts with QoxABCD. Additional support for this notion is provided by the fact that in all of the organisms in our representative set that encode *qoxABCD* (31 out of 978), *ctaM* is also present without exception. A similar tendency for co-occurrence in the same genome is observed between *ctaM* and the genes encoding cytochrome *c* oxidase subunits CoxI, -II, -III, and -IV and the alternative cytochrome *c* oxidase CoxMNOP, both *aa*_3_-type enzymes (10, 13). Furthermore, this functional association inferred by co-occurrence is strengthened by colocalization of the corresponding genes in many genomes (see Subsystem spreadsheet at reference 28). On the other hand, the *ctaM* homologues show no tendency to co-occur or to colocalize with genes that encode *bo*_3_-type ubiquinol oxidase (*cyoABCD*), *bd*-type ubiquinol oxidase (*cydABCD*), cytochrome *bc*_1_ complexes (*mcb*, *ucb*, or *cbsAB*-*soxLN*), or *cbb*_3_-type cytochrome *c* oxidase (*ccoPONQ*) (28).

The fact that only *a*-type oxidases, such as *qoxABCD* and *coxMNOP*, are linked with *ctaM* homologues by strong comparative genomics evidence implies a functional association of the CtaM protein family with heme A synthesis. Consistent with this, the co-occurrence of *ctaM* with *ctaB* (heme O synthase) has no exceptions (Fig. 6). However, 4 of the 120 genomes (3%) that contain *ctaM* do not have a *ctaA* (heme A synthase) homologue. These four exceptions are all *Archaea*, organisms in which heme A synthesis is not well understood (31, 32), and therefore do not contradict the detected functional association of the CtaM family with enzymes of heme O and A biosynthesis. In addition to a nearly absolute co-occurrence between *ctaM* and *ctaA* and/or *ctaB*, the *ctaM* genes show a strong tendency to colocalize with *ctaB* and/or *ctaA* genes within bacterial chromosomes: this is true in 79 of 120 genomes (66%) that contain *ctaM* within the set of genomes analyzed. In total, the phylogenic profile, colocalization on the chromosome, and functional analysis of the *ctaM* family clearly demonstrate that the CtaM protein functions to support the activity of *a*-type terminal oxidases in many species.

## DISCUSSION

In order to survive and proliferate in the presence of the host immune response, bacterial pathogens fine-tune their metabolism for maximal energy production and growth. The utilization of multiple terminal oxidases is one mechanism that allows pathogens to optimize metabolism in the host environment (3, 33, 34). These branched aerobic respiratory chains allow for rapid adaption to the availability of oxygen and changes in available carbon sources. The availability of oxygen is likely a major environmental cue for *S. aureus* as it transitions from a commensal organism colonizing the skin or nasopharynx to a systemic pathogen proliferating within tissue abscesses in different host organs. Certainly, the amount of oxygen on the skin and within the nose is dramatically different than in an abscess, which has recently been demonstrated to be a hypoxic environment (35). Utilizing more than one terminal oxidase allows *S. aureus* to generate the maximum amount of energy to proliferate in distinct host environments, a conclusion supported by the finding that *S. aureus* mutants restricted to a single terminal oxidase are severely impaired for colonization of the murine heart or liver (3, 4). The findings presented herein conclusively demonstrate that *S. aureus* utilizes two terminal oxidases to respire aerobically, QoxABCD and CydAB.

The *S. aureus* CydAB is resistant to micromolar concentrations (100 µM) of cyanide, distinguishing it from the cyanide-sensitive cytochrome *aa*_3_ encoded by *qoxABCD*. This profile of cyanide resistance is typical of canonical representatives of the cytochrome *bd* and cytochrome *aa*_3_ family of oxidases. However, elegant work by Voggu et al. demonstrated that nonpathogenic *S. carnosus* can grow in the presence of 1.5 mM cyanide, whereas *S. aureus* cannot (19). This is likely due to a greater sensitivity to cyanide of the cytochrome *bd* from *S. aureus*. Given the importance of CydAB to *S. aureus* virulence, a particularly intriguing notion raised from these findings is that the cyanide sensitivity of *S. aureus*, compared to that of *S. carnosus*, is an expense paid to enhance fitness during colonization of the human host.

QoxABCD is promiscuous regarding its heme cofactors and can be assembled either as an *aa*_3_-type oxidase using two equivalents of heme A or as a *bo*_3_-type oxidase using one equivalent each of heme B and heme O. In the current work, QoxABCD was constrained to assemble as the *bo*_3_-type enzyme by genetically eliminating heme A biosynthesis (Δ*ctaA* mutation), but a number of previous studies identified an *o*-type oxidase in the WT strain (14–18). Such promiscuity could possibly be an advantage, since the CtaA enzyme requires O_2_ to synthesize heme A (36). This might provide an advantage to *S. aureus* when shifting from an anoxic to oxic environment.

However, although QoxABCD is assembled as a functional enzyme in the Δ*ctaA* strain, the mutation is not benign, indicating some significant differences either in the properties of the assembled *bo*_3_ and *aa*_3_ variants of QoxABCD or in the efficiency of assembly or stability of the different forms of the enzyme. It is also possible that the inability to convert heme O to heme A reflects a different function for heme A, though none are known, or toxicity of heme O. The inability of the Δ*ctaA* strain (no heme A) to grow rapidly when CAS is substituted for glucose suggests that there is no functional respiratory oxidase present, and in the absence of a fermentable substrate (glucose), aerobic respiration is essential. Presumably, the CydAB oxygen reductase is not expressed under the conditions employed or it would be sufficient to support aerobic growth by respiration. It appears to take at least 12 h for the Δ*ctaA* strain to assemble a sufficient amount of QoxABCD to permit growth. Understanding the drastic difference in the growth phenotype will take more study, including the control of gene expression of the various respiratory components.

The elimination of the synthesis of heme A in the Δ*ctaA* strain also has consequences during infection, most prominently that this strain cannot colonize the murine liver (4). The reason for this is not clear but may reflect different concentrations of O_2_ required for the synthesis of heme A and for aerobic respiration. The growth nutrients present, as indicated by the contrast between glucose and CAS *in vitro*, may also play a role. This study validates further investigation of the requirements for QoxABCD and CydAB function as an approach to define the environmental conditions encountered by *S. aureus* during infection.

A key finding of the current work is that the assembly of a functional QoxABCD oxygen reductase requires CtaM. Our data suggest that CtaM may act as a chaperone for the insertion of heme O or heme A into QoxABCD. In support of this prediction, *ctaM* is highly conserved in respiring *Firmicutes* that also encode *qoxABCD* (Fig. 6). Additionally, heme O and heme A are still synthesized in the *ctaM* mutant (Fig. 5C), but QoxABCD is not functional (Fig. 5). This finding suggests that the hemes are not trafficked to the QoxABCD complex. However, it is currently unknown whether the hemes in the *ctaM* mutant reside within an inactive form of the QoxABCD complex or are restrained in CtaB or CtaA. The latter possibility would provide additional support for the hypothesis that CtaM acts as a heme chaperone and is required to traffic heme A or heme O to QoxABCD. The fact that *B. subtilis ctaB* and *ctaA* can be ectopically expressed and are able to synthesize heme O and heme A in *Escherichia coli*, an organism that does not express a QoxABCD oxidase, supports the possibility that the heme O and heme A are stable in the absence of an active QoxABCD complex (22). Alternatively, the presence of heme O and heme A may indicate that the function of CtaM is to assemble QoxABCD independent of heme. Further study is needed to determine the precise mechanism by which CtaM supports the assembly of QoxABCD.

## MATERIALS AND METHODS

### Bacterial strains and growth conditions.

The strains used in this study are described in Table 2. All strains are derivatives of the human clinical isolate *S. aureus* Newman or the human clinical isolate USA300 LAC JE2 adapted for laboratory use (37, 38). The Δ*cydAB* and Δ*ctaB* isogenic in-frame deletion mutants were constructed using previously described methods (39). The *cydA*, *qoxA*, *ctaA*, and *ctaM* transposon mutants were obtained from the Nebraska transposon library (NE117, NE92, NE769, and NE1084, respectively) (38). PCR confirmed that the transposon insertion mapped to the respective gene. Backcross strains of the transposon-disrupted *cydA*, *qoxA*, *ctaA*, and *ctaM* alleles into WT *S. aureus* Newman or USA300 LAC JE2 were produced via phage φ85-mediated transduction (38). The backcrossed strains were used in the studies described here. Phage φ85-mediated transduction of the *cydA* and *qoxA* transposon-disrupted alleles was used to create the Δ*ctaB* Δ*cydA* and Δ*ctaB* Δ*qoxA* double mutants. Likewise, phage φ85-mediated transduction of the *ctaA* and *ctaM* transposon-disrupted allele was also used to construct the Δ*cydAB* Δ*ctaA*, Δ*cydAB* Δ*ctaM*, Δ*qoxB* Δ*ctaA*, and Δ*qoxB* Δ*ctaM* double mutants. For complementation studies, the *ctaB* gene was PCR amplified and cloned into the pOS1 P_lgt_ plasmid using primers 5′ GCGCTCGAGATGAGCAAAGAGCATACTTTGTC 3′ and 5′ GCGGGATCCCTAGATCAAAGTAAGTAATGAAAC 3′ and the restriction enzymes XhoI and BamHI. BamHI and XhoI were also used to clone the *ctaM* gene into pOS1 P_lgt_. *ctaM* was PCR amplified using primers 5′ GCGCTCGAGATGGGCGTTCCAATTTTACCA 3′ and 5′ GCGGGATCCTTAATGACCAAATGTTGCTTTAAT 3′. Antibiotic selection of erythromycin cassette-containing resistant recipient cells was achieved with 10 µg ml^−1^ erythromycin. Antibiotic selection of strains containing the pOS P_lgt_ plasmid was achieved by using 10 µg ml^−1^ of chloramphenicol. Bacteria were routinely grown in tryptic soy broth (TSB) at 37°C. All strains were diluted 1:100 from overnight cultures into fresh TSB-containing 96-well round-bottom plates, and growth was measured by optical density at 600 nm. The PN minimal medium was prepared as previously described (38). The cells were grown overnight in TSB, washed thrice, and resuspended in PN minimal medium lacking a carbon source. Glucose and CAS were supplemented at the indicated concentrations.

**TABLE 2:** *S. aureus* strains used in this study

Strain	Relevant characteristic(s)	Reference or source
Newman strains	Wild type	37
*qoxB* mutant	*ΔqoxB*	3
*qoxA* mutant	*qoxA*::*erm*	This study
*cydAB* mutant	*ΔcydAB*	This study
*cydA* mutant	*cydA*::*erm*	This study
*ctaB* mutant	*ΔctaB*	This study
*ctaA* mutant	*ctaA*::*erm*	This study
*ctaM* mutant	*ctaM*::*erm*	This study
*ctaB cydA* mutant	*ΔctaB cydA*::*erm*	This study
*ctaB qoxA* mutant	*ΔctaB qoxA*::*erm*	This study
*qoxB ctaM* mutant	*ΔqoxB ctaM*::*erm*	This study
*cydAB ctaM* mutant	*ΔcydAB ctaM*::*erm*	This study
*qoxB ctaA* mutant	*ΔqoxB ctaA*::*erm*	This study
*cydAB ctaA* mutant	*ΔcydAB ctaA*::*erm*	This study
JE2 strains	Wild type	38
*qoxA* mutant	*qoxA*::*erm*	38
*cydA* mutant	*cydA*::*erm*	38
*ctaA* mutant	*ctaA*::*erm*	38
*ctaM* mutant	*ctaM*::*erm*	38

### Membrane preparations.

Cells were grown in LB plus 0.5% glucose for 8 h. Membranes were obtained by incubating cells with lysostaphin for 2 h at 37°C and then passing them more than five times through a microfluidizer at a pressure of 80,000 lb/in^2^. The cell extract was centrifuged at 14,000 × *g* for 10 min to remove the unbroken cells. Membranes were obtained by centrifugation at 230,000 × *g* for 4 h. Pellets, containing 30 to 40 mg/ml of protein, were resuspended in sodium phosphate buffer, pH 7.5, 50 mM NaCl, 10% glycerol, and the suspension was stored at −80°C. The NADH-reduced minus air-oxidized difference spectrum of *S. aureus* membranes was obtained at room temperature using an Agilent Technologies spectrophotometer (model 8453). Reduction was achieved by the addition of 5 mM NADH.

### Heme extraction and identification.

The hemes from *S. aureus* membranes were extracted and analyzed using high-performance liquid chromatography (HPLC) elution profiles according to established protocols (40, 41). One milliliter of the membrane suspension was solubilized by the addition of a stock solution of 20% DDM (dodecyl-β-d-maltoside) dropwise to a final concentration of 1%. The solution was incubated at 4°C for 2 h with mild agitation. The suspension was cleared by centrifugation at 230,000 × *g* for 1 h. The supernatant was mixed with 9 ml of acetone-HCl (19:1, vol/vol) and shaken on a rotary shaker for 20 min at room temperature. The mixture was centrifuged at 14,000 × *g* for 2 min, followed by the addition of 20 ml of ice-cold water and 6 ml of ethyl acetate to the supernatant. The water-ethyl acetate mixture was vortexed and centrifuged again for 10 min at 4°C. The ethyl acetate phase was recovered, and the solvent evaporated by flushing it with nitrogen gas. The residues were dissolved in 0.05 to 0.10 ml of acetonitrile and stored at −20°C. The extracted hemes were analyzed by HPLC using a Waters 2795 separation module and Waters 2996 photodiode array (PDA) detector. For heme separation, a 1-mm-inner-diameter reverse-phase C_18_ column was used with an acetonitrile (0.05% trifluoroacetic acid [TFA])-water (0.05% TFA) gradient from 50 to 100% (41). The hemes were confirmed by their molecular weights using a Waters quadrupole time of flight (Q-TOF) Ultima electrospray ionization (ESI) mass spectrometer.

### Oxygen consumption.

The oxygen concentration was monitored using a dual-channel respirometer system, model 782 from Strathkelvin Instruments, equipped with a temperature-controlled 0.5-ml electrode chamber at 37°C. The reaction mixture for this assay consisted of sodium phosphate buffer, pH 7.5, 50 mM NaCl, and 200 to 1,000 µg/ml membranes. The concentration of oxygen in the air-saturated buffer at this temperature was assumed to be 237.5 µM, and the reaction was initiated by injecting 500 µM NADH. One hundred micromoles of potassium cyanide (KCN) was added to distinguish between the cyanide-sensitive *aa*_3_ menaquinol oxidase and the cyanide-resistant *bd* menaquinol oxidase present in *S. aureus* membranes. The percentage of inhibition after the addition of KCN was calculated for each mutant.

### RNA purification and *ctaB*-*ctaM* intergenic amplification.

Bacterial RNA was harvested as previously described (42). Briefly, overnight cultures of *S. aureus* were subcultured (1:100) into TSB and cultures were harvested at late exponential phase (6 h) or stationary phase (12 h). The cells were pelleted, resuspended in LETS buffer (1 M LiCl, 0.5 M EDTA, 1 M Tris HCl, pH 7.4, 10% SDS), and lysed using mechanical bead beating. RNA was extracted using TRIzol (TRI Reagent; Sigma), chloroform precipitated with isopropyl alcohol, washed with 70% ethanol, and dissolved in distilled water. Contaminating DNA was removed with DNase I (Amersham Biosciences) treatment. RNA then was purified using the RNeasy minikit according to the manufacturer’s protocol (Qiagen). The RNA concentration and purity were measured by the optical density at 260 nm and 280 nm, respectively. For cDNA synthesis, 2 µg of RNA was left untreated (without reverse transcriptase [RT]) or was treated with Moloney murine leukemia virus reverse transcriptase according to the manufacturer’s recommendations (Promega). The region between *ctaB* and *ctaM* was PCR amplified using the following standard cycling parameters: 25 cycles of amplification with the *ctaB* forward primer 5′ ACCATCAGGCGTACTTGGTC 3′ and the *ctaM* reverse primer 5′ AATTGCCATTGGTTGGAGAC 3′.

### Bioinformatic analysis of *ctaM*.

With over 60,000 prokaryotic genomes currently available in public databases and over 15,000 more in the pipelines (www.genomesonline.org), it is not practical or possible to perform meaningful comparative analysis on all of them simultaneously. Thus, a set of diverse representative prokaryotic genomes has been developed in the SEED database as follows. The algorithm for computing molecular operational taxonomic units (OTUs) based on DNA bar code data (43, 44) was used to group ~12,600 prokaryotic genomes available in the SEED database in October 2013 into about 1,000 taxon groups. A representative genome for each OTU was selected based on the largest amount of published experimental data and the highest level of research interest within the scientific community for different microorganisms within each OTU. The resultant collection of 978 diverse eubacterial (924) and archaeal (54) genomes creates a manageable set that accurately represents the immense diversity of the over 12,000 prokaryotic organisms with sequenced genomes. Importantly, it is not skewed by an overabundance of genomes for a few microbial genera (medically or industrially important), such as *Enterobacteriaceae*, *staphylococci*, *mycobacteria*, etc.

The hypothetical DUF420 family (CtaM) has been exhaustively annotated for this set of 978 representative microbial genomes (54 archaeal and 928 eubacterial) in the SEED database (26). Functional associations for this gene family were predicted based on the patterns of co-occurrence and/or colocalization of its members with other protein families using the set of tools for comparative genome analysis available in SEED (27) within the functional and genomic contexts provided by the subsystem “Heme O and Heme A biosynthesis (with selected terminal oxidases)” (28).

## SUPPLEMENTAL MATERIAL

Figure S1  Comprehensive LC analysis of heme cofactors isolated from *S. aureus* terminal oxidase mutants and controls. (A to C) LC analysis of heme molecules isolated from the membranes of the wild type (WT) (A) and the isogenic Δ*cydA* (B) and Δ*qoxA* (C) terminal oxidase mutants. (D to F) LC analysis of heme molecules isolated from purified *aa*_3_-type quinol oxidase from *Rhodobacter sphaeroides* (D), *bd*-type quinol oxidase from *Escherichia coli* (E), and membranes isolated from *E. coli*, expressing only the *bo*_3_-type quinol oxidase (F) (Hoeser J, Hong S, Gehmann G, Gennis RB, Friedrich T. 2014. Subunit CydX of *Escherichia coli* cytochrome *bd* ubiquinol oxidase is essential for assembly and stability of the di-heme active site. FEBS Lett 588:1537–1541.) Tandem mass spectroscopy was used to identify heme D in fraction 32, heme B in fraction 37, heme A in fraction 49, and heme O in fraction 52. Download Figure S1, EPS file, 2.5 MB

Figure S2  The SCV phenotype of the Δ*ctaB* Δ*cydA* and Δ*cydAB* Δ*ctaM* double mutants can be complemented by plasmid-encoded copies of *ctaB* or *ctaM*, respectively. (A) Overnight cultures of wild-type (WT) *S. aureus* and isogenic mutants harboring an empty-vector control plasmid (pOS) or a plasmid constitutively expressing *ctaB* (p*ctaB*) were grown at 37°C in tryptic soy broth (TSB) and streaked for isolated colonies on tryptic soy agar (TSA). The Δ*qoxB* Δ*cydA* and Δ*ctaB* Δ*cydA* double mutants harboring pOS are respiration-arrested small colony variants (SCVs). (B) Overnight cultures of WT *S. aureus* and isogenic mutants were grown at 37°C in TSB and streaked for isolated colonies on TSA. The Δ*qoxB* Δ*cydA* and Δ*cydAB* Δ*ctaM* double mutants containing pOS are respiration-arrested SCVs. Download Figure S2, EPS file, 12 MB
